# Mother–Infant Relational Quality Following a NICU Stay: Investigating the Role of Maternal Childhood Experiences

**DOI:** 10.3390/ijerph22050732

**Published:** 2025-05-03

**Authors:** Corinna C. Klein, Camila A. Ferrario, Ying Yan, Nicole M. McDonald

**Affiliations:** Semel Institute of Neuroscience and Human Behavior, David Geffen School of Medicine, University of California, Los Angeles, CA,90095, USA; cferrario@mednet.ucla.edu (C.A.F.); yingyan@mednet.ucla.edu (Y.Y.); nmcdonald@mednet.ucla.edu (N.M.M.)

**Keywords:** NICU, adverse childhood experiences, benevolent childhood experiences, relational quality, mother–infant

## Abstract

A Neonatal Intensive Care Unit (NICU) stay complicates the transition to parenthood for new mothers. Women respond differently to perinatal stressors, which can impact their mental health and relationship with their new baby. Mothers’ own histories of adverse and benevolent childhood experiences can also shape their early parenting experiences. This study investigated the relationship between mothers’ adverse and benevolent childhood experiences and the observed and reported quality of interactions with their infant at 1 year following a NICU stay. Somewhat unexpectedly, we found that more maternal childhood adversity predicted less intrusive behavior and more responsiveness during a free play interaction at 12 months, while more benevolent childhood experiences predicted higher levels of observed intrusive mothering. Childhood experiences were not related to maternal perceptions of parent–child interaction quality. The length of the NICU stay was positively associated with maternal responsiveness. Findings highlight that childhood risk and protective factors may interact uniquely with a stay in the NICU, with greater adversity and a longer stay predicting more maternal responsiveness and sensitivity. Our study offers evidence that mothers can overcome their own early life challenges, and that overcoming childhood adversity may build resilience that uniquely prepares mothers for the challenge of a NICU stay.

## 1. Introduction

Maternal history of trauma and childhood maltreatment can play a significant role in influencing parenting behaviors and maternal perinatal mental health, which in turn can impact the mental and physical health of their children [1,2,3]. While the consequences of childhood trauma and adversity on an individual’s physical and mental wellbeing are by now widely accepted, increased attention is being paid to the transgenerational reverberations of childhood adversity [4,5,6]. Individuals who experienced traumatic events in childhood have higher rates of psychiatric disorders, including anxiety and depression, increased high-risk behavior (e.g., substance use), alterations in neurobiological functioning, and are at greater risk of health conditions such as heart and respiratory disease [7,8]. Childhood adversity has also been found to predict perinatal mental health [1], mother–infant dyadic quality (i.e., how the child and mother respond to, perceive, and interact with one another) [2], and child social-emotional development [9]. Childhood trauma and adversity are often assessed using the Adverse Childhood Experiences (ACEs) questionnaire, which includes experiences of abuse, neglect, and household dysfunction [10]. A meta-analysis investigating the relationship between ACEs and maternal mental health found significant associations between maternal ACEs and pre- and postnatal depression and prenatal anxiety. The meta-analysis further found that the association between ACEs and postpartum depression strengthened over the first year, becoming stronger at 12 months than at birth [1]. This finding suggests that while ACEs may not predict general baby blues or even early postpartum depressive symptoms, they can impact the duration of perinatal depression.

In addition to impacting maternal perinatal mental health, maternal ACEs are associated with lower infant developmental level (including communication, motor skills, problem-solving, and personal-social skills) at 12 months of age [11]. Differences in dyadic quality related to ACEs have also been identified. Fuchs and colleagues found that differences in observed maternal emotional availability to their infant and infant responsiveness in dyadic interactions between mothers with and without a history of maltreatment became evident at 12 months of age [2].

As the transgenerational effects of maternal childhood adversity are being increasingly understood, a growing body of research has focused on aggregating and assessing maternal childhood protective factors as well. These have been referred to as positive or benevolent childhood experiences (BCEs) [12]. BCEs, which include protective factors such as a secure attachment to a caregiver, friend, or teacher, the presence of a comforting belief system, positive experiences at school, and predictable household routines, have been found to mitigate the effects of ACEs on maternal post-traumatic stress symptomatology [12] and to predict lower peripartum depression [13]. BCEs have been theorized to directly and independently decrease mental health risk, rather than simply moderating the impact of childhood adversity and maltreatment [12,14].

Understanding the impact of risk and protective factors is particularly important for mothers experiencing perinatal stressors such as a Neonatal Intensive Care Unit (NICU) stay, which can impact early bonding and parenting. A NICU stay adds significant stress for both mother and infant and complicates the birth experience and early transition to parenthood. While a NICU stay is a stressor for all parents, it may differentially impact mothers depending on their risk and protective histories. For example, mothers with prior trauma exposure or early separation from their own caregivers may have heightened stress responses or reactions to the early separation from an infant created by a NICU stay. Exposure to childhood trauma heightens vulnerability to the subsequent development of psychopathology in response to later life traumatic exposure and stressors [15,16]. Conversely, mothers with protective factors, such as strong social support or a history of secure relationships, may be better able to adapt to the inherent challenges of a NICU stay.

Regardless of maternal history, a NICU stay involves unique stressors that complicate the postnatal period. Early separation from a baby can affect parental responsiveness and early bonding. Fostering secure attachments involves early bonding practices such as skin-to-skin contact, which are often limited by medical concerns in the NICU environment [17]. The highly medicalized environment of the NICU combined with the physical separation can create feelings of disconnection, isolation, and anxiety for parents [18]. Parental stress and anxiety associated with perinatal complications can disrupt parents’ attunement to their infant and interfere with parents’ emotional response to their child [19,20]. These early bonding disruptions can strain the parent–child relationship by increasing parents’ anxiety related to their caregiving abilities [21]. Additionally, many NICU graduates have specialized care needs due to medical and developmental considerations, which add unique stress to early parenting.

The purpose of this study was to assess the relationship between maternal childhood adversity and protective factors, and mother–infant relational quality in families who experienced a NICU stay, a significant perinatal stressor. Relational quality includes interactive behaviors that form the basis of a child’s early social environment and can shape subsequent social-emotional development, such as maternal sensitivity (how responsive and attuned a mother is to her infant’s cues), maternal intrusion (moments of overriding or disrupting the infant’s behaviors), infant responsiveness (the infant’s verbal or affective engagement with and reaction to the mother’s bids), and dyadic reciprocity (the infant and mother’s mutual responsiveness to one another) [22]. The current study draws from attachment theory, developmental psychology, and the literature on the transgenerational transmission of trauma. While the impact of ACEs and BCEs on parenting and early mother–infant relational quality has been investigated [2,23,24,25,26,27,28], it has not been evaluated in the context of a NICU stay. Existing literature indicates that ACEs are associated with a higher likelihood of a NICU stay [29], but has not clarified how these maternal histories may transmit in the context of this perinatal stressor. This study expands on the existing literature by illuminating how maternal history of risk and protective factors interacts with a significant perinatal stressor in shaping the mother–child relationship.

This study aimed to: (1) investigate the relationship between maternal ACEs and BCEs and observed and reported mother–infant relational quality at 12 months, (2) assess whether the degree of medical complication (length of NICU stay) corresponds with observed or reported relational quality, and (3) examine whether ACEs and the length of the NICU stay predict observed mother–infant dyadic quality at 12 months following a NICU stay. We hypothesized that more ACEs and fewer BCEs would be associated with less sensitive parenting, more intrusive parenting, less infant responsiveness, and less dyadic reciprocity. We anticipated that mothers who had experienced more ACEs and fewer BCEs would report more dysfunctional parent–child interactional dynamics. Finally, we expected that a longer NICU stay would be associated with lower dyadic quality, more intrusive parenting, less infant responsiveness, and higher reported dyadic dysfunction.

## 2. Methods

### 2.1. Participants and Procedures

The data for this study were extracted from a broader longitudinal study, entitled Neurodevelopment and Early Social-Emotional Trajectories in NICU Graduates (NESTING). The focus of the NESTING study was to understand early social and brain development in high-risk NICU graduates. Participants were patients at the High-Risk Infant Follow-up (HRIF) Clinic at the University of California, Los Angeles, recruited by research associates who provided them with written and verbal information about the study. Interested families were contacted by a research associate to participate in a screening interview, then scheduled for their first visit if they qualified for the study. All infants who participated in this study had a history of NICU hospitalization and met the criteria for the California HRIF program as defined by California Children’s Services, which include neurological issues, cardiac issues, increased medical risk, preterm birth (<32 weeks), and/or low birth weight (≤1500 g).

Data were collected for this study from 2021 to 2024. Infant participants were enrolled by 6 months of age. A total of 137 eligible families were informed of the study, with 53 (39%) enrolling. Participating families took part in a HIPAA-compliant remote visit via Zoom when their infant was 6 months and 9 months old and hybrid in-person/remote visits at 12 and 24 months. The primary focus of this study was the 12-month visit, given its relative proximity in time to the perinatal stressors and medical complications. Additional information was collected during the 6-month (medical history) and 24-month (maternal history) visits. The visits were scheduled based on chronological age for infants born full-term and adjusted age based on expected due date for infants born preterm (gestational age < 37 weeks). Parents of the infants participated in clinical interviews with trained clinicians and completed online questionnaires (see Section 2.2. Measures below) and additional information about infant medical history was gathered from their medical records. Clinicians and research assistants were closely supervised by the study’s principal investigator (senior author) through weekly meetings and ongoing check-ins. All parents provided informed consent to participate in the study and to allow the research team to access their child’s medical records. This study has been approved by the University of California, Los Angeles Medical Institutional Review Board 3 (MIRB3).

Forty-five mother–infant dyads provided data at the 12-month visit and 36 mothers self-reported ACEs/BCEs at the 24-month visit. Data on childhood experiences at 24 months were missing for varied reasons (i.e., mother declined questionnaire, *n* = 1; father-only data available, *n* = 3; missed 24-month visit, *n* = 4). One twin was excluded to ensure the independence of data points, resulting in 44 mother–infant dyads. Table 1 summarizes the participating families’ demographic data.

### 2.2. Measures

#### 2.2.1. Medical and Demographic Data

Interviews with mothers were conducted at each child’s 6-month visit by a trained research associate to collect infant and mother demographic and medical data. Medical history was also confirmed via review of each infant’s medical records. Medical data gathered included length of NICU stay, gestational age, and infant birth weight. Maternal medical history included delivery type, multiparity, and method of conception.

Degree of medical complication: The length of the NICU stay was used as a proxy for the severity of medical complication. The length of the NICU stay was found to be a good predictor of 12-month development in a previous study with this sample [30].

#### 2.2.2. Maternal Childhood Experiences

##### Adverse Childhood Experiences

The Adverse Childhood Experiences (ACEs) survey is a 10-item “yes or no” questionnaire, with a “prefer not to answer” option included, that collects information about the adversity an individual has faced between 0 and 18 years old. The questionnaire includes seven categories based on the original ACEs questionnaire [10] about maltreatment during childhood (i.e., psychological, physical, and sexual abuse) and dysfunctional household experiences, such as exposure to domestic violence, substance abuse, mental illness, incarceration, neglect or parental divorce/separation. Scores range from 0 to 10. The questionnaire was administered to parents during their infant’s 24-month visit (*n* = 36).

##### Benevolent Childhood Experiences

The Benevolent Childhood Experiences (BCEs) survey is a 10-item “yes or no” questionnaire, also with a “prefer not to answer” option included, that collects information about the positive experiences an individual had in the first 18 years of their life [12]. The questionnaire gauges the participant’s experiences with internal and relational safety and security (e.g., “Did you have at least one caregiver with whom you felt safe?”), predictable quality of life (e.g., “Did you have a predictable home routine, like regular meals and a regular bedtime?”), and interpersonal support (e.g., “Did you have at least one teacher who cared about you?”). Scores can range between 0 and 10. The questionnaire was also administered to parents at the 24-month visit (*n* = 36).

#### 2.2.3. Observed Dyadic Quality

Parent–child interactions were recorded via Zoom while they engaged in a free play session at home for a total of 5 min at the 12-month visit. Five minutes of free play is consistent with prior research using the same coding system to observe mother–infant interactions [31,32,33] and minimizes the time burden for caregivers and infants. Instructions were given to mothers to: “Play with your baby as you normally do.” A trained examiner monitored the interaction with their camera off. Free play interactions were available for all 44 mother–infant dyads included in the current study.

##### Free Play Coding

Parent–child interactions were coded offline based on the Coding of Interactive Behavior system developed by Ruth Feldman [34]. In this study, two codes measuring parenting behavior (acknowledging and overriding), one code related to child behavior (child response), and one code assessing dyadic behavior (dyadic reciprocity) were analyzed. Behavior was globally rated on a scale from 1 to 5 in half-point increments. Maternal acknowledging evaluated the mother’s vocal, physical, and emotional expressions of sensitivity to the child, with a focus on responses to the child’s cues. Overriding assessed the mother’s intrusiveness via disruptions to the child’s behavior, attempts to redirect the child’s attention, and ignoring the child’s age-appropriate signals. Child response measured the child’s active (verbal or non-verbal) response to the parent’s social bids, such as smiles or eye contact. Dyadic reciprocity reflects the degree of “give-and-take”, mutual, synchronous interaction between mother and child.

##### Reliability

Coding was completed by two research assistants who were trained to reliability with the second and senior authors, the latter of whom was initially trained by Dr. Ruth Feldman and her team. Coders were blind to other participant data. Coders overlapped on 34% of interactions to assess reliability. Intraclass correlations (ICCs; single measures, absolute agreement) revealed high reliability for each code: maternal acknowledging (0.93), maternal overriding (0.90) child response (0.91), and dyadic reciprocity (0.89).

#### 2.2.4. Reported Dyadic Interaction Quality

The Parent–Child Dysfunctional Interaction (P-CDI) scale from the Parenting Stress Index Short Form (PSI-4-SF) [35] was used as a measure of parent perception of the quality of interactions with their infant, with higher scores indicating poorer interaction quality. This scale is part of a larger 36-item measure, of which it comprises 12 items. The PSI-4-SF has excellent reliability and internal consistency. It was collected at the 12-month visit and was available for 43 mothers.

### 2.3. Data Analysis Plan

Analyses were conducted using SPSS 29 and 30. Descriptive data were analyzed to assess the range and levels of risk and protective factors in the sample, dyadic quality, and parent perception of interactive quality. Data were checked for outliers, skewness, and kurtosis. To investigate the relationship between maternal ACEs and BCEs and parent–child observed (CIB codes) and reported (P-CDI) relational quality at 12 months, bivariate correlations were run between variables. Due to the skewness of ACEs and BCEs, Spearman’s rho was calculated. To investigate whether the degree of medical complication corresponds with observed or reported relational quality at 12 months, Pearson’s correlations were calculated between length of NICU stay and CIB ratings, and between length of NICU stay and P-CDI scores. Two separate regressions were then run with ACES and length of NICU stay as independent variables, and maternal acknowledging and overriding as dependent variables.

## 3. Results

### 3.1. Descriptive Statistics

Descriptive statistics were calculated to assess the range of adverse and benevolent childhood experiences in this sample of moms who experienced a NICU stay. Mothers reported low levels of adverse childhood experiences overall (*M* = 1.64, *SD* = 1.94), with 64% experiencing at least one ACE and 16.7% reporting four or more ACEs. Mothers reported high levels of BCEs overall (*M* = 8.06, *SD* = 1.62), with 61.1% of mothers reporting nine or more BCEs. Additional descriptive information is included in Table 2 and Table 3.

### 3.2. Aim 1: Investigate the Relationship Between Maternal ACEs and BCEs and Parent–Child Relational Quality at 12 Months

Spearman’s rank correlations were computed to assess the relationship between ACEs and BCEs and CIB ratings. Unexpectedly, mothers with fewer ACEs displayed more overriding during play with their child. Similarly, mothers with higher BCEs showed lower levels of responsiveness to their children’s cues and more overriding behavior. ACEs and BCEs were not correlated with child social response or dyadic reciprocity (see Table 4). Spearman’s rank correlations were also computed to assess the relationship between ACEs and BCEs and P-CDI ratings. Maternal ratings of interaction quality were not significantly correlated with their number of ACEs or BCEs.

### 3.3. Aim 2: Investigate Whether Length of NICU Stay Corresponds with Relational Quality at 12 Months

Pearson’s correlations were calculated between length of NICU stay and 12-month CIB ratings and between length of NICU stay and P-CDI scores. Mothers who experienced longer NICU stays showed higher levels of responsiveness to their child during play, *r*(42) = 0.332, *p* = 0.028. No other significant correlations were found between length of NICU stay and observed (CIB; *rs* = −0.063–0.034) or parent-reported interaction quality (P-CDI; *r* = 0.109).

### 3.4. Aim 3: Do ACEs and the Degree of Medical Complication Predict Maternal Behavior at 12 Months Following a NICU Stay?

Two separate multiple linear regressions were run to predict maternal overriding and maternal acknowledging from ACEs and length of NICU stay. Residuals were examined for normality, linearity, and homoscedasticity, and multicollinearity was assessed. The first regression model explained significant variability in maternal overriding, *F*(2, 33) = 4.274, *p* = 0.022, *R*^2^ = 0.206. Examination of individual predictors indicated that the number of ACEs was a significant predictor of maternal overriding while the length of the NICU stay was not. Likewise, the second regression model explained significant variability in maternal acknowledging, *F*(2, 33) = 4.906, *p* = 0.014, *R*^2^ = 0.229. Both individual predictors were significant in this model. Regression results are presented in Table 5.

## 4. Discussion

The current study investigated the relationship between maternal early life risk and protective factors (ACEs and BCEs) and dyadic quality in the context of one significant perinatal stressor, a NICU stay. We investigated the degree to which maternal adverse and benevolent childhood experiences and the length of the NICU stay corresponded with the observed and perceived quality of mother–child interactions at 12 months.

### 4.1. Rates of ACEs and BCEs

We found that rates of ACEs in our sample were similar to those found in community samples of perinatal women [9,36]. However, our sample had a smaller proportion of mothers with four or more ACEs than has previously been found among mothers with a child in the NICU. In a prior study of NICU mothers, 32.8% reported at least four ACEs compared to 16.7% in our sample [37]. Another study that followed women from their first prenatal appointment to 6 weeks postdelivery found that higher ACE exposure is related to much higher likelihood of a NICU admission (nine times higher odds with six or more ACEs) [29]. Only one mother in our sample endorsed all 10 BCEs, while in previous studies of pregnant women, 44% [38] and 28% [12] endorsed all 10, though our average number of about 8 BCEs was comparable to previous findings.

Limited variability in BCEs scores could be due to ceiling effects; the original BCEs questionnaire, which we used, has been revised to include additional items that are less frequently endorsed due to potential ceiling effects associated with the original scale [39]. While our sample was demographically and socioeconomically diverse, it may have selected for parents with less childhood adversity than is typically found among NICU families. For instance, infants who were not in a stable caretaking situation were generally unable to sign up for a longitudinal study. Additionally, the sample included a relatively low proportion of families with lower family income, possibly due to life stressors that interfered with lower income families’ capacity to commit to a research study. Given that ACEs and BCEs were collected at families’ 24-month visits, it is also possible that we retained fewer families with more maternal childhood adversity.

### 4.2. Childhood Risk and Protective Factors and Dyadic Quality

Contrary to our hypotheses and in contrast to previous findings, more childhood adversity in our sample was associated with higher levels of observed maternal sensitivity and lower levels of maternal intrusiveness. It is possible that in the context of a NICU stay, the impact of ACEs on parenting behavior is different, or that childhood adversity actually helped mothers develop resilience that enabled them to better respond to perinatal stressors. This interpretation is speculative and should be replicated in future research. We also found that ACEs and the length of the NICU stay independently predicted maternal observed acknowledging, such that more ACEs and a longer NICU stay predicted more responsive mothering. Typically, we would expect the cumulative impact of early life adversity combined with increased perinatal stress to negatively impact parenting approach and dyadic quality. A previous study found less observed sensitivity and emotional availability to their 12-month-old in mothers with a history of abuse compared to mothers without [2]. Notably, Fuchs and colleagues’ study excluded preterm babies with APGAR scores less than seven, making it very different from our sample of infants with more significant medical histories, whose average APGAR scores at 1 min were 5.35. Parenting a medically complex infant presents unique challenges and can elicit different parenting behaviors characterized by concern, uncertainty, or increased caution. In fact, mothers of infants at high neurological risk have been found to use less voiced speech when interacting with their infants [40]. Another study found less maternal sensitivity among mothers with a history of maltreatment in mother–infant dyads at 16 months, mediated by parenting stress [41]. While previous research suggests that maternal childhood adversity may contribute to less sensitive mothering, perinatal and birth-related stressors may not have the same impact. In fact, a meta-analysis of studies assessing maternal sensitivity among parents of preterm babies found no differences between mothers of babies born preterm versus those born full term [42].

Our study was unique in that it investigated the combined impact of early life adversity and perinatal stress on dyadic quality. While preliminary, our findings suggest that some level of adverse experiences in childhood may actually build resilience, preparing these mothers to confront the challenge of a NICU stay. This finding echoes the literature on post-traumatic growth, which indicates that many people experience positive psychological change following traumatic experiences [43]. Positive psychological change catalyzed by trauma can include an increased appreciation of life, a shift in perspective, closer relationships, and a belief in one’s personal strength [43]. Each of these changes could benefit a parent experiencing a NICU stay or medical stressor, particularly the belief in one’s capacity to handle challenges.

While post-traumatic growth is one possible explanation for the increased sensitivity of mothers with higher ACEs in our study, it is also possible that these mothers are more attentive to or preoccupied with their infant’s early adversity, having experienced childhood adversity themselves. Their histories may have primed them to be sensitive to their child’s distress or discomfort, resulting in more observed sensitivity and less intrusion in the context of a NICU stay and potential ongoing medical and developmental challenges. Mothers with greater experiences of adversity may have also had more exposure to their own interventions and psychotherapy, supporting the development of sensitive responding and their capacity for attunement. Overall, the moms in our sample showed relatively high levels of responsiveness and low levels of intrusive behavior. It is possible that the free play situation was less suited toward capturing challenges with maternal responsiveness that may arise in the face of a more stressful situation, such as when needing to soothe an upset baby. Future studies may assess maternal response to child distress through scenarios such as removing a preferred toy or brief separation from a parent.

More benevolent childhood experiences in this sample were associated with higher levels of maternal intrusive behavior (overriding). Although overriding is generally considered a more intrusive and less optimal form of interacting with one’s infant, it is possible that in this sample, overriding reflected necessary maneuvering of the infant’s body or leading of the play necessitated by developmental delays or other characteristics unique to children who have experienced a NICU stay. If this were the case, however, it would be expected that length of NICU stay may also be associated with overriding, which it was not. It is possible that a generally positive childhood characterized by supportive experiences may result in a higher sense of competence and confidence in one’s parenting, whereby parents interact with their child in response to their instincts and intuitions rather than with measured awareness of their infant’s cues as guides. More research is certainly needed to understand and replicate these findings and to illuminate whether they would be replicated in a larger sample with and without a NICU experience.

### 4.3. Length of NICU Stay and Dyadic Quality

A longer NICU stay in our sample predicted higher levels of maternal acknowledging behavior. While the stress and anxiety associated with a long hospitalization at birth could result in parenting characterized by post-traumatic stress symptoms, such as greater detachment or dissociation, in this sample it seemed to actually result in greater attentiveness to infant cues. There are several potential mechanisms that may explain this finding, including maternal adaptation to the NICU stay, the specific needs of the NICU infant, or elements of the NICU environment. Regarding maternal adaptation, it is possible that mothers who have experienced long separations from their infant develop compensatory attentiveness and closeness in interactions with their infant. Alternately, it is possible that infants with longer stays have more developmental or medical needs that demand increased attention and responsiveness. With respect to the NICU environment, many NICUs integrate family-based or dyadic interventions, providing more bonding encouragement and support to new mothers and fostering sensitive parenting [44]. Even in NICUs where these interventions have not been implemented, the setting involves exposure to specialists and providers who offer infant education or model infant care. These elements of the NICU environment may help explain why a longer NICU stay would entail more direct parenting support and strategies for sensitive responding, resulting in more sensitive maternal behavior later on. This explanation is speculative, however, as we do not have information on particular interventions to which parents in this study were exposed.

A previous longitudinal study that assessed changes in observed maternal behavior over time following a NICU stay did not find that the number of days in the NICU related to any scale of mothering behavior during the hospitalization, though their sample comprised exclusively of preterm babies [45]. Additionally, their sample stayed in the NICU for an average of 30.31 days (*SD* = 27.16), compared to 55.89 days (*SD* = 45.43) in the current sample. It is possible that impacts on mothering become more pronounced following longer hospitalizations or in relation to other medical concerns. Since our sample was heterogeneous in the reason for a NICU stay, a longer NICU stay could mean more medical complications, more prematurity, and potentially lower developmental abilities at 12 months [30]. The most common qualifying condition in our study was very or extreme prematurity (45.54%), followed by hypoxic–ischemic encephalopathy (HIE) or other significant neurological issue (20.5%). Maternal sensitivity following hospital discharge has not been found to differ based solely on prematurity [42], but a longer length of NICU stay could indicate extreme prematurity, which can result in later developmental delays that elicit increased maternal sensitive responding. In fact, longer NICU stays were found to predict lower developmental abilities at 12 months in a different study of this sample [30]. More sensitive parenting could be a response to developmental delays. HIE or neurological issues at birth can also result in different medical and developmental trajectories that elicit varied parenting responses. Higher maternal acknowledging in this context may be a response to heightened attentiveness given the infant’s physical or developmental needs or may be influenced by exposure to early intervention services accessed after discharge.

### 4.4. Limitations and Future Directions

This study was limited by the relatively small and heterogeneous sample and will need to be replicated with a larger cohort. A sample size of 50 NICU graduates was initially planned, with consideration of both feasibility and power analyses that were grounded in the primary aims for the larger study. Given the modest sample size, the current study should be considered preliminary and must be replicated within a larger sample to confirm the findings. Findings could be specific to this sample or to the time frame studied. The infants in this study were also born in the latter half of the COVID-19 pandemic, adding unusual stressors and additional medical considerations to their birth and hospitalization experiences. It is possible that some of this study’s findings may be attributable to the unique context of the pandemic. Attrition bias may also have impacted results since ACEs and BCEs were not collected until the 24-month visit. It is possible that families with more childhood adversity were less likely to attend their 24-month visit. Self-report questionnaires are also inherently limited in that families may have been hesitant to truthfully report their childhood adversity exposure, although this would similarly affect all studies using these measures.

There are also inherent benefits and limitations to brief observed interactions in the home setting. Home-based interactions are more convenient for participants, particularly mothers with young children. Home- and clinic-based parent–child interactions have also been found to reflect similar interactional behaviors [46]. While home-based video interactions have the benefit of a more naturalistic setting, they do not afford the environmental consistency and controls that lab-based observations do. Therefore, it is possible that external environmental factors may have impacted mother–infant play interactions. Additionally, visibility limitations may have resulted in missed subtleties of engagement by mother or child. Finally, while five-minute free play interactions are common in prior research, a longer observation or one that stressed the dyad may have yielded additional information about dyadic patterns.

Finally, our study assessed cumulative ACEs, which include multiple types of adversity (e.g., abuse, neglect, household dysfunction). While cumulative ACEs have been found to contribute to increased health risk, it is possible that the types of adversity contribute to maternal behavior and response to perinatal stress in distinct ways. For example, ongoing emotional neglect may shape maternal sensitivity differently than an individual instance of physical or sexual abuse. Similarly, parental incarceration or divorce likely impacts maternal behavior uniquely. Future research with a larger sample should disaggregate childhood adversity to assess whether particular types of childhood adversity (e.g., physical or sexual abuse vs. emotional neglect or maltreatment) contribute uniquely to later parenting following a NICU stay.

### 4.5. Conclusions

Overall, this study offers unique initial insights into how maternal childhood experiences interact with a NICU stay to impact later parenting behaviors and perceptions. We unexpectedly found that mothers with higher levels of childhood adversity were more responsive to their child’s cues in a play situation, suggesting that overcoming early challenging experiences may build resilience when faced with a stressor in adulthood, like a child’s NICU stay. Our results indicate that less attuned maternal behaviors should not be assumed based on maternal history, and that history, current environmental stress, and ongoing experiences interact to shape later mother–child interactions.

## Figures and Tables

**Table 1:** Participant characteristics (*n* = 44).

Mother	*M* (*SD*)
Maternal age at conception (years)	33.43 (7.10)
	N (%)
Method of conception	
Spontaneous	34 (77.3%)
Doctor-assisted	10 (22.7%)
First pregnancy	15 (34.1%)
Type of delivery	
Vaginal	12 (27.3%)
C-section	32 (72.7%)
Maternal race	
White	22 (50.0%)
Black/African American	10 (22.7%)
Multiracial	1 (2.3%)
Asian/Pacific Islander	8 (18.2%)
Other	3 (6.8%)
Maternal ethnicity	
Hispanic	16 (36.4%)
Not Hispanic	28 (63.6%)
Maternal education	
High school or less	6 (13.6%)
Some college	13 (29.5%)
College degree	13 (29.5%)
Graduate/professional degree	12 (27.3%)
Family income	
Under $25,000	6 (13.6%)
$25,000–$49,999	7 (15.9%)
$50,000–$74,999	9 (20.5%)
$75,000–$124,999	6 (13.6%)
$125,000+	14 (31.8%)
Marital status	
Single parent	6 (13.6%)
Other/separated	2 (4.5%)
Married	31 (70.5%)
Living together	5 (11.4%)
**Infant**	*M* (*SD*)
Gestational age (weeks)	33.19 (5.25)
Infant birth weight (grams)	1914.48 (989.48)
Length of NICU stay (days)	55.89 (45.43)
APGAR at 1 min	5.35 (2.61)
APGAR at 5 min	7.28 (2.07)
	N (%)
Infant sex	
Female	22 (50.0%)
Male	22 (50.0%)
Infant race	
White	24 (54.5%)
Black/African American	10 (22.7%)
Multiracial	2 (4.5%)
Asian/Pacific Islander	7 (15.9%)
Infant ethnicity	
Hispanic	20 (45.5%)
Not Hispanic	24 (54.5%
HRIF Primary Qualifying Condition	
Preterm <32 weeks	20 (45.54%)
VLBW ≤1500 g, (32 weeks +)	6 (13.6%)
HIE/other significant neuro (>1500 g, ≥32 weeks)	9 (20.5%)
Cardiac (no major neuro, >1500 g, ≥32 weeks	4 (9.1%)
Other (no major neuro/cardiac, >1500 g, ≥32 weeks)	5 (11.4%)

Two participants were missing data on family income. Race was missing for one Hispanic infant. VLBW: Very low birth weight. HIE: Hypoxic–Ischemic Encephalopathy. Infants were placed into only one HRIF group based on priority order above (e.g., 31-week infant who weighed 1300 g placed in preterm group). Infants in “Other” group had severe respiratory issues, pulmonary hypertension, hernia, necrotizing enterocolitis, sepsis, and severe hypoglycemia. Length of NICU stay ranged from 5 to 176 days.

**Table 2:** Mean, SD, and range for maternal ACEs, BCEs, P-CDI, and CIB scales.

Scale	N	Minimum	Maximum	Mean	SD
Adverse Childhood Experiences	36	0.00	7.00	1.64	1.94
Benevolent Childhood Experiences	36	3.00	10.00	8.06	1.62
Parent–Child Dysfunctional Interaction	43	11.00	32.00	18.37	5.91
Maternal Acknowledging	44	1.00	5.00	4.19	1.01
Maternal Overriding	44	1.00	5.00	1.93	1.04
Child Response	44	1.00	5.00	3.89	0.99
Dyadic Reciprocity	44	1.00	5.00	3.83	0.994

ACEs: Adverse Childhood Experiences, BCEs: Benevolent Childhood Experiences, P-CDI: Parent–Child Dysfunctional Interaction, CIB: Coding of Interactive Behavior.

**Table 3:** Frequency of ACEs and BCEs (*n* = 36).

	N	Percent of Sample
Number of ACEs		
0	13	36.0%
1	11	30.6%
2	1	2.8%
3	5	13.9%
4 or more	6	16.7%
Number of BCEs		
10	1	2.8%
9	21	58.3%
8	5	13.9%
7	4	11.1%
6 or fewer	5	14.0%

ACEs = Adverse Childhood Experiences, BCEs = Benevolent Childhood Experiences.

**Table 4:** Correlations between ACEs, BCEs, and reported and observed dyadic quality (*n* = 36).

Spearman’s Rank	Maternal Acknowledging	Maternal Overriding	Child Response	Dyadic Reciprocity	Parent–Child Dysfunctional Interaction
ACEs	0.356 *	−0.515 **	0.120	0.257	0.121
BCEs	−0.557 **	0.351 *	−0.055	−0.123	−0.268

** Correlation is significant at the 0.01 level. * Correlation is significant at the 0.05 level.

**Table 5:** Linear regressions predicting observed maternal overriding and acknowledging.

	*b*	SE	Beta	t	*p*
Overriding					
Intercept	2.373	0.286		8.298	<0.001
ACEs	−0.245	0.084	−0.454	−2.924	0.006
Length of NICU stay	0.000	0.004	0.000	0.001	0.999
Acknowledging					
Intercept	3.408	0.288		11.819	<0.001
ACEs	0.196	0.084	0.354	2.319	0.027
Length of NICU stay	0.008	0.004	0.323	2.113	0.042

## Data Availability

The data presented in this study are available on request from the corresponding author (ccklein@mednet.ucla.edu).
